# Identification and functional characterization of the extremely long allele of the serotonin transporter-linked polymorphic region

**DOI:** 10.1038/s41398-021-01242-9

**Published:** 2021-02-11

**Authors:** Tempei Ikegame, Yosuke Hidaka, Yutaka Nakachi, Yui Murata, Risa Watanabe, Hiroko Sugawara, Tatsuro Asai, Emi Kiyota, Takeo Saito, Masashi Ikeda, Tsukasa Sasaki, Mamoru Hashimoto, Tomohisa Ishikawa, Minoru Takebayashi, Nakao Iwata, Chihiro Kakiuchi, Tadafumi Kato, Kiyoto Kasai, Miki Bundo, Kazuya Iwamoto

**Affiliations:** 1 Department of Neuropsychiatry, Graduate School of Medicine, The University of Tokyo, Tokyo, Japan; 2 Department of Neuropsychiatry, Faculty of Life Sciences, Kumamoto University, Kumamoto, Japan; 3 Department of Molecular Brain Science, Graduate School of Medical Sciences, Kumamoto University, Kumamoto, Japan; 4 Department of Psychiatry, Kansai Rosai Hospital, Hyogo, Japan; 5 Department of Psychiatry, Fujita Health University School of Medicine, Toyoake, Japan; 6 Laboratory of Health Education, Graduate School of Education, The University of Tokyo, Tokyo, Japan; 7 Department of Psychiatry, Graduate School of Medicine Osaka University, Osaka, Japan; 8 Department of Psychiatry and Behavioral Science, Graduate School of Medicine, Juntendo University, Tokyo, Japan; 9 Laboratory for Molecular Dynamics of Mental Disorders, RIKEN CBS, Wako, Japan; 10 International Research Center for Neurointelligence (WPI-IRCN), The University of Tokyo Institutes for Advanced Study (UTIAS), The University of Tokyo, Tokyo, Japan

**Keywords:** Medical genetics, Epigenetics in the nervous system

## Abstract

*SLC6A4*, which encodes the serotonin transporter, has a functional polymorphism called the serotonin transporter-linked polymorphic region (5-HTTLPR). The 5-HTTLPR consists of short (S) and long (L) alleles, each of which has 14 or 16 tandem repeats. In addition, the extralong (XL) and other rare alleles have been reported in 5-HTTLPR. Although they are more frequent in Asian and African than in other populations, the extent of variations and allele frequencies (AFs) were not addressed in a large population. Here, we report the AFs of the rare alleles in a large number of Japanese subjects (*N* = 2894) consisting of two cohorts. The first cohort (case-control study set, CCSS) consisted of 1366 subjects, including 485 controls and 881 patients with psychosis (bipolar disorder or schizophrenia). The second cohort (the Arao cohort study set, ACSS) consisted of 1528 elderly subjects. During genotyping, we identified 11 novel 5-HTTLPR alleles, including 3 XL alleles. One novel allele had the longest subunit ever reported, consisting of 28 tandem repeats. We named this XL_28-A._ An in vitro luciferase assay revealed that XL_28-A_ has no transcriptional activity. XL_28-A_ was found in two unrelated patients with bipolar disorder in the CCSS and one healthy subject in the ACSS who did not show depressive symptoms or a decline in cognitive function. Therefore, it is unlikely that XL_28-A_ is associated with psychiatric disorders, despite its apparent functional deficit. Our results suggest that unraveling the complex genetic variations of 5-HTTLPR will be important for further understanding its role in psychiatric disorders.

## Introduction

The serotonin transporter (5-HTT) encoded by *SLC6A4* is a key molecule that regulates serotonergic neurotransmission at the synaptic cleft, affecting emotions and stress responses. Therefore, numerous studies have focused on elucidating the pathophysiological implications of psychiatric disorders. Animal studies using constitutive *Slc6a4* knockout mice showed increased anxiety-like phenotypes in various behavioral tests^1–3^. Furthermore, human studies indicate that dysregulation of 5-HTT in the serotonergic system is implicated in the emotional and behavioral disturbances of psychiatric disorders, including anxiety, depression, bipolar disorders (BD), schizophrenia (SZ), and autism^4^.

*SLC6A4* has a functional polymorphism (serotonin transporter-linked polymorphic region, 5-HTTLPR) in its promoter region. 5-HTTLPR consists of two major alleles: the short (S) and the long (L), each of which has 14 or 16 repeat units^5^, composed of 20–23 bp of highly homologous sequence units^6^, with the S allele exhibiting weaker transcriptional activity than the L allele^7–9^. On the basis of the dichotomous classification, myriad case-control association studies have been performed. Most of the studies and several meta-analyses claimed that the S allele confers sensitivity to environmental stress, which in turn increases vulnerability to anxiety and depressive symptoms^10–12^, although it remains controversial^13,14^, and the recent largest meta-analysis and case-control studies failed to replicate this finding^15,16^.

In addition to the major S and L alleles, 5-HTTLPR has a number of rare variants. To date, extrashort (XS) repeats (11–13 repeats)^17–19^, 15 repeats^5,19^, and extralong (XL) repeats (17–24 repeats)^5,18–26^ have been reported. These rare variants showed a higher frequency in Asian and African populations than in European and Native American populations^19,27^. However, despite the extensive genetic studies on 5-HTTLPR so far, the extent of variations and allele frequencies (AFs) have not been addressed in detail in a large population.

Here, we report the patterns and AFs of 5-HTTLPR rare variants in the Japanese population in detail. Through the genotyping of two cohorts, we examined 5-HTTLPR in a total of 2894 Japanese subjects. The first cohort (the case-control study set, CCSS) consisted of 1366 subjects, including 485 controls and 881 patients with major psychosis (BD and SZ). The second cohort (the Arao cohort study set, ACSS) consisted of 1528 subjects who are community-dwelling elderly individuals. In total, we identified 11 novel 5-HTTLPR alleles. One of the novel alleles had the longest subunit ever reported, consisting of 28 tandem repeats. We named this extremely long allele XL_28-A._ Interestingly, a luciferase reporter assay confirmed that XL_28-A_ has no transcriptional activity. XL_28-A_ was found in two unrelated patients with BD in the CCSS and one subject in the ACSS, who did not show depressive symptoms or a decline in cognitive functions. Therefore, it is unlikely that XL_28-A_ is associated with psychiatric disorders, despite its apparent functional deficit. Our results suggest that unraveling and interpreting the complex genetic variations of 5-HTTLPR will be important for further understanding its role in psychiatric disorders.

## Materials and methods

### Study subjects

We used two independent Japanese DNA sample sets to examine the genetic variations of 5-HTTLPR. The first sample set (the CCSS) consisted of 1366 subjects, including 485 controls (CTs) and 881 patients with major psychosis (450 BD and 431 SZ). The details of the CCSS were previously reported^28^. All patients were diagnosed according to the DSM-IV (Diagnostic and Statistical Manual of Mental Disorders, Fourth Edition) criteria by experienced psychiatrists. CTs were collected based on voluntary recruitments from employees, students, and their friends and were interviewed by senior psychiatrists. All CTs were confirmed to have met the following criteria: (i) no current or past Axis-I psychiatric or physical diagnoses and (ii) no first-degree relatives with SZ or BD. For both patients and CTs, we excluded subjects with a history of current and past neurological illnesses, traumatic brain injuries, electroconvulsive therapy, and substance abuse.

The second sample set (the ACSS) consisted of 1528 elderly subjects. The ACSS addresses community-dwelling Japanese individuals aged over 65 who live in Arao city in Kumamoto prefecture in Japan. This is a part of the Japan Prospective Studies Collaboration for Aging and Dementia. Among the endpoints available, we utilized the Mini-Mental State Examination (MMSE)^29^ and Geriatric Depression Scale (GDS)^30^ for this study. These data are collected for the purpose of examining the baseline dementia or depression level at the start of the project.

The ethics committees of Kumamoto University and other collaborative research organizations approved this study. All subjects received a detailed description of this study and provided written informed consent.

### DNA extraction and genotyping

Extraction of genomic DNA from peripheral blood cells (PBCs) and genotyping in the CCSS were previously reported^28^ and are briefly summarized in the Supplementary Methods. In the ACSS, genomic DNA was extracted from PBCs with a QIAamp DNA Blood Mini QIAcube Kit (Qiagen, Hilden, Germany) using a QIAcube (Qiagen). 5-HTTLPR was amplified with the following primers: FWD 5′-CTTTGCGTTTTCTGTTGCCC−3′ and REV 5′-GGAGGCCAGGAACGATAGGA−3′. PCR amplification was performed in a total volume of 20 µL solutions containing the following compositions: 10 µL of 2× PCR buffer for KOD FX (TOYOBO, Osaka, Japan), 4 µL of 12 mM dNTP mix, 0.6 µL each of 10 µM primers, 2 U of KOD FX (TOYOBO) and 10 ng of genomic DNA. The thermocycling conditions were as follows: an initial cycle of 2 min at 94 °C, followed by 30 cycles of 10 s at 98 °C and 1 min at 68 °C. All PCR amplicons were analyzed by MultiNA (SHIMAZU, Kyoto, Japan) and by bidirectional Sanger sequencing with 5′-GGCGTTGCCGCTCTGAATGC−3′ or 5′-CAGGGCGGGGACCGCAAGGT−3′. In some amplicons, we additionally performed TA cloning using a TOPO cloning kit (Invitrogen, Carlsbad, CA) followed by Sanger sequencing analysis of individual colonies.

### Plasmid construction

The target 5-HTTLPR variant was amplified with the following primers: FWD 5′-AAgagctcGGTGAAATTCCCAAGCTTGTTG-3′ with the *Sac*I site (lowercase letters) and REV 5′AActcgagTTCTGGTGCCACCTAGACGC-3′ with the *Xho*I site (lowercase letters). PCR amplification was performed in a total volume of 25 µL solutions containing the following compositions: 2.5 µL of 10 × PCR buffer for KOD-Plus-Neo (TOYOBO), 1.5 µL of 25 mM MgSO_4_ (Promega, Madison, WI, USA), 0.5 µL of 10 mM dNTP mix (Invitrogen), 2 µL each of 10 µM primers, 0.5 U of KOD-Plus-Neo (TOYOBO) and 50 ng of genomic DNA. The thermocycling conditions were as follows: (1) an initial cycle of 2 min at 94 °C, (2) 33 cycles of 10 s at 98 °C and 30 s at 72 °C. The PCR fragments were gel-purified with a MinElute Gel Extraction Kit (Promega) followed by 3′ A-attachment with TaKaRa Ex Taq (Takara, Tokyo, Japan) and cloned into the PCR 2.1 vector using the TOPO cloning kit. The *Sac*I and *Xho*I fragments were subcloned into the pGL4.10 firefly luciferase reporter vectors (Promega). The single bacterial colony was cultured in a large volume, and the plasmid vector was purified with an Endotoxin-free plasmid DNA purification kit (NucleoBond Xtra EF purification system, Macherey-Nagel GmbH, Düren, Germany), followed by ethanol precipitation. All constructs were bidirectionally sequenced to check for proper orientation, sequence specificity and the absence of artificial mutations.

### Luciferase reporter assay

Undifferentiated rat raphe-derived RN46 cell lines (Sigma-Aldrich, St. Louis, MO, USA) grown under standard conditions were cotransfected with 2 µg of each luciferase reporter construct, 200 ng of the pGL4.73 *Renilla* luciferase reporter vector (Promega) as an internal control, and 200 ng of pCMV-EGFP vector as a positive control using an electroporator (NEPA21, NEPA GENE, Tokyo, Japan). Twenty-four hours after transfection, cells were harvested and lysed, and luciferase activity was measured using a Dual Luciferase Assay System (Promega) and a GloMax™ 96 Microplate Luminometer (Promega) following the manufacturer’s protocol. Each construct was transfected three times in each of three assays for a total of 9 independent transfections. Firefly/*Renilla* ratios were calculated in each transfection and normalized to the mean ratio of blank control vectors, pGL4.10 (Promega).

## Results

### Identification of novel variants of 5-HTTLPR in the CCSS

**Fig. 1:**
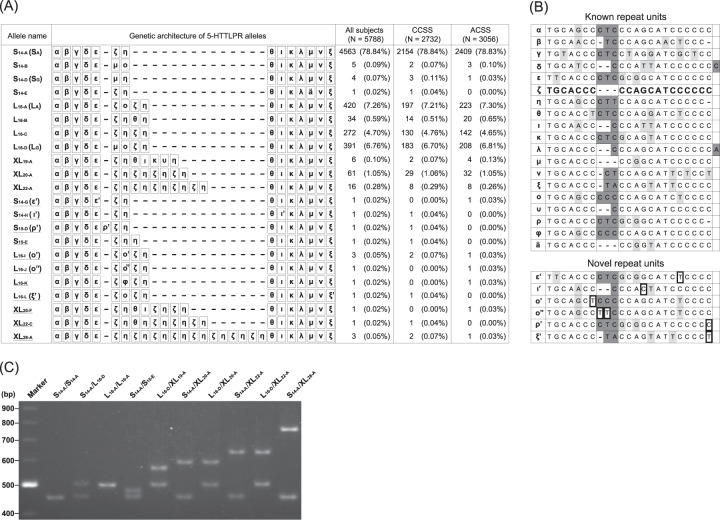
Nucleotide sequences of repeat units and the genetic architecture of 5-HTTLPR alleles. **A** Names, genetic architecture, and allele frequencies of 5-HTTLPR alleles identified in this study. The allele names refer to the comprehensive summary of 5-HTTLPR alleles^6^, and individual repeat units are identified by the Greek-letter nomenclature introduced by Nakamura et al^5^. Novel alleles are underlined. The total number of alleles and their allele frequencies (%) are given. ﻿CCSS: case-control study set, ACSS: Arao cohort study set. **B** Structure of repeat units. Each repeat unit is assigned a Greek letter following the nomenclature of Nakamura et al. The nucleotide sequence of ζ is shown in bold. Nucleotide substitutions are highlighted in light gray, and insertions are in dark gray compared to ζ. Dark boxes in novel repeat units indicate nucleotide substitutions or insertions compared to the original repeat units. **C** Representative 5-HTTLPR genotypes in agarose gel electrophoresis. The lengths of the PCR amplicons are as follows: S_14-A_, 457 bp; S_15-E_, 479 bp; L_16-A_, 499 bp; L_16-D_, 499 bp; XL_19-A_, 567 bp; XL_20-A_, 583 bp; XL_22-A_, 625 bp; and XL_28-A_, 751 bp.

We previously performed genotyping of 5-HTTLPR in 1366 subjects of the CCSS and reported the epigenetic role of major alleles, including Asian-specific L_16-C_^28^. In this study, we examined rare alleles (AF < 5%) and found three known S variants (S_14-B_, S_14-D_ and S_14-E_), one known L variant (L_16-B_), and three known XL alleles (XL_19-A_, XL_20-A_, and XL_22-A_). In addition, we identified seven novel alleles (S_14-H_, S_15-D_, S_15-E_, L_16-I_, L_16-L_, XL_22-C_, and XL_28-A_) (Fig. 1A, B). Representative alleles were visualized by electrophoresis on agarose gel (Fig. 1C).

### Structure of the novel 5-HTTLPR alleles in the CCSS

Among the novel alleles, S_14-H_, L_16-I_, L_16-L_, and S_15-D_ were likely to be generated from base substitutions in the known repeat units. S_14-H_ has a T/C substitution at the 12th base position in ι of the common S_14-A_ allele. L_16-I_ has a C/T substitution at the 7th base position in ο of the common L_16-A_ allele. L_16-L_ has a C/T substitution at the 22nd base position in ξ of L_16-A_. On the other hand, S_15-D_ was generated by single-base cytosine insertion at the end of ρ of S_15-A_^6^. Each of these mutated repeat units in S_14-H_, L_16-I_, L_16-L_, and S_15-D_ was described as ι’, ο’, ξ’, and ρ’, respectively (Fig. 1B).

The other three novel alleles, S_15-E,_ XL_22-C_, and XL_28-A_, were likely to be generated from novel arrangements of the known repeat units. S_15-E_ contains a tandem duplication of a single η repeat compared with S_14-A_. XL_22-C_ has a replacement of ξ with θ at the place of the 8th repeat unit in XL_22-A_. XL_28-A_ has tandem duplications of eight ζ-η units compared with the one ζ-η unit allele S_14-A_.

### Allele frequencies of the rare variants in psychosis in the CCSS

AFs with regard to diagnostic groups are listed in Supplementary Table 1. Using Fischer’s exact test, none of the rare variants (AF < 5%), including novel alleles, showed significant deviation in patients compared to controls (*p* ≥ 0.05). Nonetheless, among the seven novel alleles, XL_28-A_ was found in two unrelated patients with BD and not in controls. Considering that XL_28-A_ is the longest allele so far identified, we performed a functional assay of this allele.

### Functional characterization of XL_28-A_ using a luciferase reporter assay

We examined the promoter activities of XL_28-A_ and four known alleles (S_14-A_, L_16-C_, XL_20-A,_ and XL_22-A_) using a luciferase reporter assay in the rat raphe-derived RN46 cell line. Each allele has a different number of ζ-η tandem repeats from one to eight (Fig. 1A, Table 1). We observed that the promoter activity was maintained as the number of tandem repeats was up to 5, with a slight decrease in longer repeats. However, we found that the promoter activity of XL_28-A_ with 8 tandem repeats was at the same level as a blank control vector, indicating that XL_28-A_ has no apparent promoter activity (Table 1).

**Table 1:** Promoter activities of XL_28-A_ and other alleles with a different number of ζ-η.

allele	number of ζ-η	promoter activity^a^	*p*-value^b^
S_14-A_	1	1.55 ± 0.05	<0.001
L_16-C_	2	1.49 ± 0.15	<0.001
XL_20-A_	4	1.26 ± 0.06	0.024
XL_22-A_	5	1.37 ± 0.08	<0.001
XL_28-A_	8	1.01 ± 0.08	0.999

^a^Ratio to blank control vector, pGL4.10 (mean ± SD).

^b^Dunnett test with pGL4.10 as reference.

### Validation of XL_28-A_ in the general population

We then extensively screened for the presence of XL_28-A_ and other rare alleles using the ACSS (*N* = 1528). Through genotyping in the ACSS, we found six novel alleles (S_14-G_, L_16-I_, L_16-J_, L_16-K_, XL_20-F_ and XL_28-A_). Among them, two alleles, L_16-I_ and XL_28-A_, were already found in the CCSS, and the other four were newly identified in this cohort (Fig. 1A). By combining the subjects of the CCSS and the ACSS, none of the rare alleles showed significant deviation in patients compared to controls by Fisher’s exact test (*p* ≥ 0.05).

### Structure of the novel 5-HTTLPR alleles (S_14-G_, L_16-J_, L_16-K_, and XL_20-F_) in the ACSS

S_14-G_ has a C/T substitution at the 19th base position in ε of S_14-A_. L_16-J_ has two consecutive C/T substitutions at the 8th and 9th base positions in ο of L_16-A_. These mutated repeat units were described as ε’ and ο” (Fig. 1B). L_16-K_ has a replacement of φ with ο or η at the place of the 7th repeat unit in L_16-A_ or L_16-C_. XL_20-F_ has an insertion of a θ-ι unit between the 7th and 8th repeat units in XL_18-A_^6^.

### Relationship between the novel 5-HTTLPR alleles and either MMSE or GDS in elderly subjects

**Fig. 2:**
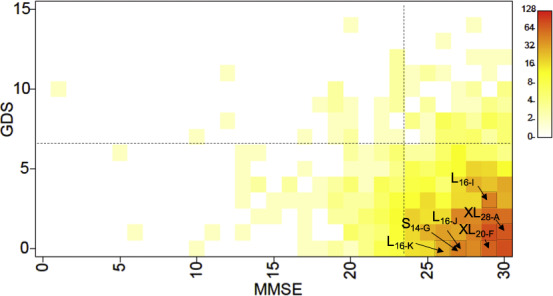
Assessment of novel 5-HTTLPR alleles on the MMSE and GDS in elderly subjects. Scores of the MMSE and GDS are plotted. Colors indicate the total number of subjects. Arrows indicate the scores of subjects with novel alleles. The dotted line indicates the general thresholds of the MMSE (< 23) and GDS (> 8). Scores above (MMSE) and below (GDS) the thresholds indicate that the subject has no decline in cognitive function or no tendency toward depression. All indicated subjects had a novel allele that was heterozygous with S_14-A_.

Among the endpoints available in the ACSS, we utilized MMSE and GDS. A subject harboring the XL_28-A_ showed that both scores were at normal levels and within the normal variation in the ACSS population (Fig. 2). In addition, we observed that the other five novel alleles also did not show the possibility of major depressive disorder or dementia.

## Discussion

In this study, we identified a total of 11 novel rare alleles and obtained the AFs of known rare alleles of 5-HTTLPR in two independent Japanese cohorts. Among the reported rare alleles, we did not observe 11, 17, 21, and 24 repeat alleles, which were found in other ethnic populations^18,24,25^. We also did not observe 13 and 18 repeat alleles, which were reported in Japanese subjects^19,31,32^. Considering the sample sizes, our study achieved the most accurate and comprehensive estimation of the rare alleles in the Japanese population.

### Genetic sequence and architecture of novel allelic variants

Among the 11 novel alleles, 8 were estimated to originate from either L_16-A_ or S_14-A_. In addition, variable duplication of ζ-η subunits was involved in the other three alleles. XL_28-A_ has eight ζ-η unit repeats inside the genomic rearrangement region, while S_14-A_ has only one ζ-η unit. The exact duplications of the ζ-η unit have been identified in L_16-C_, XL_18-A_, XL_20-A_, XL_20-E_, and XL_22-A_. It would be reasonable to expect the presence of other intermediate numbers of repeat units with tandem duplications of ζ-η, such as 24 and 26.

### XL_28-A_ lost its promoter activity

Our promoter assay revealed that the extremely long XL_28-A_, which harbors 8 ζ-η tandem repeats, had no promoter activity, while other alleles with a smaller number of repeats maintained their activities (Table 1). A previous study demonstrated that lymphoblastoid cell lines (LCLs) derived from American-African females homozygous or heterozygous with XL alleles, which are 81 bp longer than the L allele and presumably XL with 20 repeats, represent higher expression levels compared to those with SS or LL^33^. This result raised the hypothesis that an increased length of the 5-HTTLPR allele is associated with increased promoter activity. Our contrary result would be explained by the fact that the expression level in the previous study was affected by other regulatory polymorphisms, such as StIn2^34^ and rs25531 SNP^35^, in *SLC6A4*. Another possibility would be that we only examined ζ-η tandem repeats, and the XL alleles consisting of other units may exhibit a length-dependent increase in promoter activity.

Using the transcription factor binding profile database JASPAR (http://jaspar.genereg.net/), we found that the ζ-η tandem repeat has presumptive binding sites of the TFAP2 family, consisting of five members: TFAP2α, TFAP2β, TFAP2γ, TFAP2δ, and TFAP2ε. It is known that TFAP2 proteins have essential roles in neuronal development^36,37^. In humans and mice, TFAP2β exists ﻿in the cerebellum, midbrain, medulla, and pons throughout adulthood^38^ and represses the promoter activity of *SLC6A4* via the TFAP2 binding site at the rs25531 SNP of unit μ in L_16-D_^39^. Given that the presumptive TFAP2 binding site we found in this study is located over the ζ and η units, the accumulation of ζ-η tandem repeats might lead to the repression of the promoter activity of *SLC6A4*.

### Pathophysiological role of the XL alleles

In the CCSS, we found no evidence of an association of the AF of XL alleles with BD or SZ. Contrary to our initial expectation, our extensive genotyping in the ACSS showed that the AF of XL_28-A_ was not associated with psychiatric disorders. In addition, a subject with XL_28-A_ did not show an apparent decline in cognitive function, nor was there a signature of depression in the elderly subjects. The absence of promoter activity of 5-HTTLPR, therefore, has no apparent effect on phenotypes related to psychiatric disorders. This would be explained by complementation by other *cis-*regulatory elements, such as StIn2^34^ and rs25531 SNP^35^, in *SLC6A4* and/or by another 5-HTTLPR allele. Therefore, it would be interesting to pursue the functionality by generating cells with homozygous XL_28-A_.

Our results suggest that the identification and interpretation of numerous 5-HTTLPR genetic variations will be important for understanding the role of 5-HTTLPR in psychiatric disorders. We have previously reported that psychiatric patients harboring low-activity alleles showed altered DNA methylation at specific CpG sites in *SLC6A4* and altered amygdala volume^28^. Integrative analysis of DNA methylation and 5-HTTLPR as well as brain structure will be worth studying in the future.

## Supplementary information

Supplementary Materials
