# Supplementary material for: Identification and functional characterization of the extremely long allele of the serotonin transporter-linked polymorphic region
Source: Transl Psychiatry. 2021 Feb 11;11:119. doi: 10.1038/s41398-021-01242-9 (PMC7878853; doi:10.1038/s41398-021-01242-9)
Supplement: Supplementary file 1 — Supplementary Materials [file 41398_2021_1242_MOESM1_ESM.docx]

**Supplementary Materials**

**Supplementary Methods**

**Determination of 5-HTTLPR in the CCSS**

DNA extraction from whole blood was performed using a Wizard Genomic DNA purification kit (Promega) following the manufacturer’s instructions. The concentration and purity of genomic DNA were determined using a spectrophotometer (Nanodrop, Wilmington, DE, USA). To use as the template for 5-HTTLPR genotyping PCR, each genomic DNA sample was diluted to a final concentration of 50 ng/µl with distilled water.

5-HTTLPR was amplified with the following primers: FWD 5’- GGTGAAATTCCCAAGCTTGTTG-3’ and REV 5’- TTCTGGTGCCACCTAGACGC-3’. PCR amplification was performed in a total volume of 20 µL solutions containing the following compositions: 4 µL of 5 x Promega Flexi buffer (Promega), 4.8 µL of 5 M betaine (Sigma-Aldrich), 1.6 µL of 25 mM MgCl_2_ (Promega), 0.8 µL of 10 mM dNTP mix (Invitrogen), 4 µL each of 10 µM primers, 0.75 U of GoTaq Hot Start DNA Polymerase (Promega) and 50 ng of genomic DNA. The thermocycling conditions were as follows: 1) an initial cycle of 2 min at 94°C; 2) 33 cycles of 30 s at 94°C, 30 s at 65°C and 30 s at 72°C; and 3) 1 cycle of 10 min at 72°C. The genotypes were confirmed by electrophoresis on a 2.5% agarose gel.

| Supplementary Table 1. Allele frequencies in diagnostic groups. | | | |
| --- | --- | --- | --- |
|  | CCSS | | |
| Allele name | CT (N = 970) | BD (N = 900) | SZ (N = 862) |
| S_14-A_ (S_A_) | 734 (75.67%) | 727 (80.78%) | 693 (80.39%) |
| S_14-B_ | 1 (0.1%) | 0 (0%) | 1 (0.12%) |
| S_14-D_ (S_G_) | 2 (0.21%) | 1 (0.11%) | 0 (0%) |
| S_14-E_ | 0 (0%) | 0 (0%) | 1 (0.12%) |
| L_16-A_ (L_A_) | 76 (7.84%) | 60 (6.67%) | 61 (7.08%) |
| L_16-B_ | 7 (0.72%) | 5 (0.56%) | 2 (0.23%) |
| L_16-C_ | 61 (6.29%) | 32 (3.56%) | 37 (4.29%) |
| L_16-D_ (L_G_) | 66 (6.8%) | 60 (6.67%) | 57 (6.61%) |
| XL_19-A_ | 1 (0.1%) | 0 (0%) | 1 (0.12%) |
| XL_20-A_ | 15 (1.55%) | 7 (0.78%) | 7 (0.81%) |
| XL_22-A_ | 3 (0.31%) | 4 (0.44%) | 1 (0.12%) |
| S_14-G_ (ε’) | 0 (0%) | 0 (0%) | 0 (0%) |
| S_14-H_ (ι’) | 1 (0.1%) | 0 (0%) | 0 (0%) |
| S_15-D_ (ρ’) | 0 (0%) | 1 (0.11%) | 0 (0%) |
| S_15-E_ | 0 (0%) | 1 (0.11%) | 0 (0%) |
| L_16-I_ (ο’) | 2 (0.21%) | 0 (0%) | 0 (0%) |
| L_16-J_ (ο”) | 0 (0%) | 0 (0%) | 0 (0%) |
| L_16-K_ | 0 (0%) | 0 (0%) | 0 (0%) |
| L_16-L_ (ξ’) | 1 (0.1%) | 0 (0%) | 0 (0%) |
| XL_20-F_ | 0 (0%) | 0 (0%) | 0 (0%) |
| XL_22-C_ | 0 (0%) | 0 (0%) | 1 (0.12%) |
| XL_28-A_ | 0 (0%) | 2 (0.22%) | 0 (0%) |
